# Preparation of a novel palladium catalytic hydrogel based on graphene oxide/chitosan NPs and cellulose nanowhiskers†

**DOI:** 10.1039/c8ra06623j

**Published:** 2018-09-24

**Authors:** Samira Ashiri, Ebrahim Mehdipour

**Affiliations:** Faculty of Science, Department of Chemistry, Lorestan University Khorramabad Iran mehdipour.e@lu.ac.ir

## Abstract

The present paper focuses on the synthesis of a novel hydrogel support by combining polysaccharides (chitosan NPs and dialdehyde cellulose nanowhiskers) and graphene oxide nanosheets to obtain a biocompatible material for catalytic applications. The hydrogel was synthesized *via* green chemistry processes and used as a support to prepare Pd nanoparticles. Finally, the hydrogel@Pd NPs was employed as the catalyst in the Mizoroki–Heck reaction to generate new C–C bonds. SEM analysis indicated that the hydrogel has macroporous morphology, which is in good correlation with its high crosslinking degree. The as-synthesized nanocomposite hydrogel exhibits beneficial properties such as ease of separation and excellent recyclability for at least six cycles without considerable loss in its activity. The yields of the products range from 81% to 98%. Additionally, this study provides the possibility to perform the Mizoroki–Heck reaction with aryl chloride in the presence of the as-prepared catalyst.

## Abstract 2

Pd NPs dispersed on a polysaccharide-based hydrogel as a novel catalyst show high catalytic activity and recyclability for coupling reactions.

## Graphical abstract

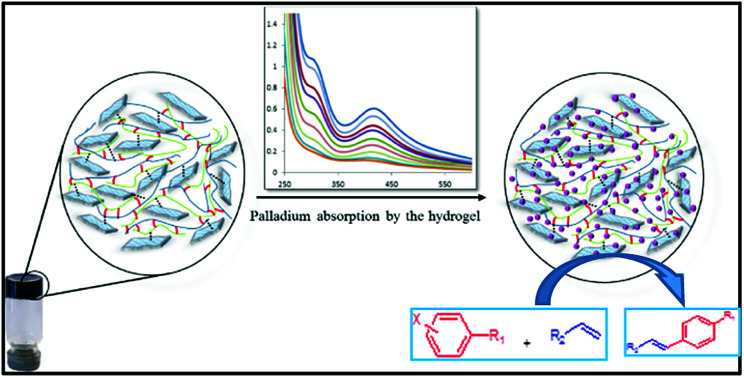

## Introduction

In recent years, the concepts of “Green Chemistry” have gained considerable importance. Green chemistry is the combined research in environmental protection and chemistry, and is extended to all aspects of synthetic chemistry.^1^ Therefore, the design of chemical products and use of hazardous substances are an emerging important subject in chemical research and industry due to health, economic, and environmental concerns. Recently, a large number of industrial and academic groups have investigated the development and application of green catalysts to reduce or eliminate the use of dangerous substances.^2^ Homogeneous catalysts have various advantages such as improved performances, high selectivity and easy optimization *via* modification with metals and ligands. However, the difficulty of catalyst separation and probabilistic toxicity caused by the remaining metal species in the reaction limit their use in synthetic and industrial applications.^3^ Therefore, catalyst systems that not only exhibit high selectivity and activity (homogeneous systems), but are also easy to recover and separate (heterogeneous systems) are necessary.^4^ The disadvantages can be reduced by using homogeneous complexes connected to insoluble solid supports. Owing to its many benefits such as easy isolation, and reduction in by-products and cost, heterogenization of catalysts to solid supports can provide the chance for recycling catalysts from reaction mixtures.^5^ Nanocatalysts, through preserving the desirable attributes of both homogeneous and heterogeneous catalysts, eliminate the gap between these two types of catalysts.^6^ Among the heterogeneous nanocatalysts, metal nanoparticles have emerged as a new class of catalysts for organic processes. Nevertheless, the major problem in using metal nanoparticles as catalysts is their aggregation.^7^ Thus, different materials such as clays, surfactants, alumina, polymers, and hydrogels have been widely used as solid supports to immobilize metal nanoparticles.^8^

The past two decades have witnessed a rapid development of hydrogels as novel materials in both the academic and industrial domains.^9,10^ Hydrogels are three-dimensional (3D) polymeric networks that are usually cross-linked (either physically or/and chemically) to render the network insoluble.^11^ Furthermore, the structural properties of hydrogels can allow the *in situ* synthesis of nanoparticles.^12,13^ Due to the presence of functional groups in the hydrogel network, such as –COOH, –OH, –SH, –SO_3_H and –NH_2_, the hydrogel allows water and other liquids to penetrate into its structures and cause different metal ion loading into its matrices.^14^ Therefore, hydrogels are suitable for use as solid supports to prepare and immobilize metal nanoparticles. Nowadays, polysaccharide-based hydrogels such as hyaluronic acid, chitin, starch, chitosan^15^ and alginate have been investigated as promising materials.^16^ Compared with hydrogels produced using synthetic polymers, natural polysaccharide-based hydrogels have various advantages such as environmental friendliness^17^ and greater accessibility. Cellulose, starch and chitin are the most abundant organic molecules on Earth, and are widely considered in many research fields.^18^ Recently, to improve the mechanical properties of polysaccharide-based hydrogels and add new functional features, nanomaterial and polymer hydrogel hybrids have been widely studied, particularly in the catalysis field.^19^ The modification of polysaccharide-based hydrogels by graphene can be useful since hydrogels not only prevent restacking of individual graphene nanosheets but also disperse metal NPs uniformly on their surface. Moreover, they can generate a three-dimensional (3D) porous structure to improve transfer and mass diffusion during catalytic reactions.^20–22^ Carbon–carbon bond formation is one of the most conducive single operational methods to construct various bioactive compounds and natural products. It has also been industrially important for the synthesis of organic frameworks over the past few decades. Thus, to date, there have been many efforts to develop more efficient catalytic systems by designing and synthesizing suitable catalysts.^23^ Since palladium nanoparticles (NPs) do not need ligands and often have recovery capabilities, they are known as green catalysts. However, the stability and reactivity of this catalyst system are highly dependent on the substrates that are often used to immobilize the Pd NPs. In this study, for the first time, hydrogels as solid supports were used to immobilize palladium nanoparticles.^24^ Herein, we attempted to design and synthesize hydrogels based on polysaccharides (chitosan NPs and dialdehyde cellulose nanowhiskers) and graphene oxide to create suitable solid supports for adsorbing and immobilizing Pd NPs. The hydrogel composite containing Pd NPs was prepared as a catalyst for use in the Mizoroki–Heck reaction, and its catalytic activity was investigated. In addition, the importance of using green solvents has permeated all aspects of synthetic chemistry, and the Mizoroki–Heck reaction is no exception to this rule. In most cases, cross-coupling reactions preferentially occur in highly dipolar aprotic solvents such as DMAc, DMF, and NMP. Although these solvents deliver high performances for coupling reactions, their impact on the environment must be considered.^25^ In this study, a mixture of ethanol and water was used to optimize the solvent properties.

## Experimental

### Materials and equipment

In this study, commercial cotton linters were used as the cellulose material. Chitosan (75–85% degree of deacetylation, *M*_w_:190 000–310 000 Da), graphite, sodium tungstate dehydrate (Na_2_WO_4_·2H_2_O), phosphoric acid (H_3_PO_4_), sodium nitrate (NaNO_3_), sulfuric acid (H_2_SO_4_), potassium permanganate (KMnO_4_), hydrochloric acid (HCl), sodium hydroxide (NaOH), methanol, dimethylformamide (DMF), and hydrogen peroxide (H_2_O_2_) were purchased from Sigma-Aldrich. Aryl halides, olefins, and thin layer chromatography (TLC) plates were purchased from Merck. FT-IR spectra of the products were recorded on a Bruker-Tensor320 spectrometer. For the determination of particle size and size distribution, dynamic light scattering (DLS) was performed on a Malvern Zetasizer equipped with a photodiode detector at an orientation of 90°. Thermogravimetric analysis analyses were performed on an STA 409 apparatus (Linei) at a heating rate of 10 °C min^−1^ under nitrogen gas. XRD patterns for the compounds were recorded using a Siemens diffractometer with Cu-K radiation at 35 kV. An LEO 440i scanning electron microscope under vacuum at an operating voltage of 10 kV was used to study the morphology and surface structure of the samples. Dried samples were used for the SEM experiment, which were coated with a thin layer of gold by sputtering for 15 s. For recording the absorption spectra of the sample in solution, a Shimadzu UV-vis 1650 PC spectrophotometer was used with a cell of 1.0 cm path length. Inductively coupled plasma optical emission spectroscopy (ICP-OES) (Perkin-Elmer 7300D model spectrometer) was used for determining the amount of Pd adsorbed by the hydrogel. ^1^HNMR spectra for samples dissolved in CDCl_3_ and DMSO were recorded on a Bruker DRX-400 spectrometer operated at 400 MHz.

### Methods

#### General procedure for the preparation of graphene oxide (GO)

Graphene oxide (GO) was prepared from graphite powder using a modified Hummer's method according to the reported procedure in the literature (ref. 26).

#### General procedure for the preparation of chitosan NPs

Nanochitosan was prepared *via* an ionic-gelation method with sodium tungstate dihydrate. Chitosan (0.5 g) was dissolved in 2% acetic acid solution and then, 5 mL sodium tungstate dehydrate (concentration of 2 g L^−1^) was added dropwise to 50 mL chitosan solution. The product was washed several times with distilled water to eliminate any sodium tungstate dihydrate, and then centrifuged. The chitosan NPs were separated by centrifugation and then freeze-dried.

#### General procedure for the preparation of cellulose nanowhiskers (CNW)

Cellulose nanowhiskers were prepared *via* acid hydrolysis. For this purpose, 4 g cotton linter was cut into small fragments and dispersed in a 2% NaOH solution. Then, the suspension was stirred for 12 h at rt, filtered and washed with water to neutral pH. After washing, the resulting cellulose fibers were air-dried and then added to H_2_SO_4_ (65%) (ratio of fibers : acid solution 1 : 8.75 g mL^−1^) in a 50 °C water bath for one hour. After treatment, the fiber slurry turned into a milky colloid suspension, which was then centrifuged. The sample was continually washed until neutral pH was obtained. The product was freeze-dried. The obtained powder CNWs were stored at 5 °C for further characterization.^27^

#### Synthesis of oxidized cellulose nanowhiskers (DCNW)

Dialdehyde cellulose nanowhiskers (DCNW) were prepared according to previous research with slight modification.^28,29^ Initially, 1 g nanocrystal cellulose solution was dispersed in 3 mL water and sonicated in an ultrasonic bath cleaner for 20 minutes. Subsequently, a few drops of sodium hydroxide solution were added until pH ten was reached. Then, 0.0008 g of copper sulfate was added as a catalyst. Subsequently, hydrogen peroxide (H_2_O_2_) was slowly added to the reaction mixture at 30 °C. After the addition of hydrogen peroxide was complete, 10 drops of NaOH were added and the mixture was then stirred for 30 min. Sodium bisulfite was added dropwise until neutral pH was obtained. The product was collected by filtration and washed several times with water. The resulting sediment was dried under vacuum at 40 °C. To determine the carbonyl content in DCNW, Cu^2+^ titration was performed according to the reported procedure, and the carbonyl content obtained was 0.301 mmol g^−1^.^30^

#### General procedure for the preparation of the hydrogels

**Scheme 1:**
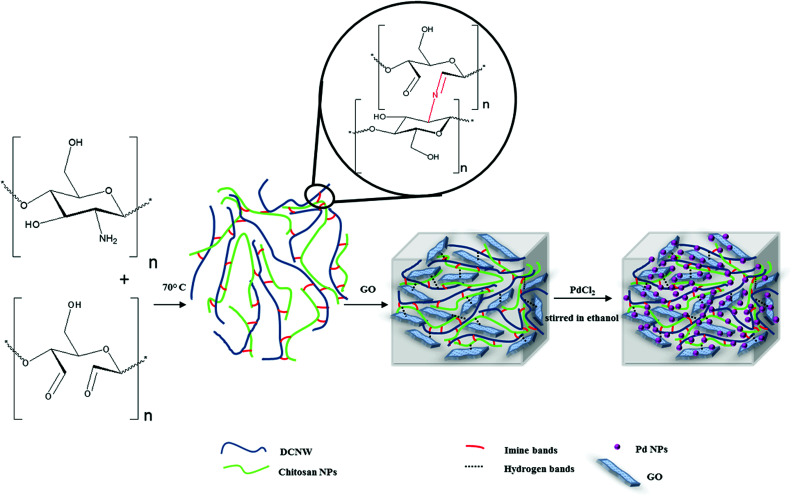
Schematic of the method for the synthesis of the hydrogels.

A solution of DCNW (0.5 g) was prepared by sonication for 1 h until the mixture became uniform. The mixture was placed in an oil bath at 40 °C and stirred for 24 h. Then, a certain amount of chitosan NPs (0.5 g in 3 mL acetic acid 2%) was slowly added to the mixture reaction and stirred heavily at 60–70 °C. The degree of substitution for dialdehyde cellulose whiskers according to the ninhydrin assay^31^ was 57.6%. A turbid solution was obtained. Finally, 0.25 g graphene oxide was added to the turbid solution, which was stirred at room temperature for 35 min. Then, the obtained hydrogel was freeze-dried. The method of synthesizing the hydrogels is shown in Scheme 1.

#### General procedure for the preparation of hydrogel@Pd

To prepare hydrogel@Pd, 0.5 g of PdCl_2_ was added to water (50 mL) and stirred at room temperature for 24 h. Then, 0.5 g of the hydrogel was added and stirred. During the reaction, to observe the amount of palladium adsorbed, the UV-Vis absorption of the solution was measured at a specific time interval (10 min). Then, to ensure the complete conversion of Pd(ii) to Pd(0), the reaction mixture was stirred in ethanol overnight. After stirring with ethanol, the obtained black solid was dried under vacuum.^32^ The loading of Pd in the hydrogel was determined *via* ICP analysis. The concentration of palladium was 21.57 wt%.

#### General procedure for preparative Mizoroki–Heck reactions

To a flask, a mixture of hydrogel@Pd (0.2 g), olefin (2.2 mmol), aryl halide (1 mmol) and K_2_CO_3_ (1.5 mmol) in 2 mL EtOH/H_2_O (1 : 1) was added. The mixture was refluxed (85 °C) for the appropriate time under aerobic conditions. The progress was monitored by thin-layer chromatography (EtOAc/hexane, 20 : 80). After completing the reaction, the mixture was cooled to room temperature and the catalyst separated by simple filtration and dialysis with EtOH/H_2_O for 2 h. The reaction solution was diluted with EtOAc (15 mL). The organic layer was dried over anhydrous Na_2_SO_4_ and concentrated under reduced pressure. The residue was purified by recrystallization from EtOH and H_2_O. The catalyst was dried for reuse at 70 °C for 2 h.

## Results and discussion

In this study, the as-synthesized hydrogel showed the ability to adsorb palladium metal, which was applied as the catalyst for the coupling reaction. To obtain better insight into the synthesis process and catalytic activity of hydrogel@Pd, various analyses were performed on this nanocomposite.

**Fig. 1:**
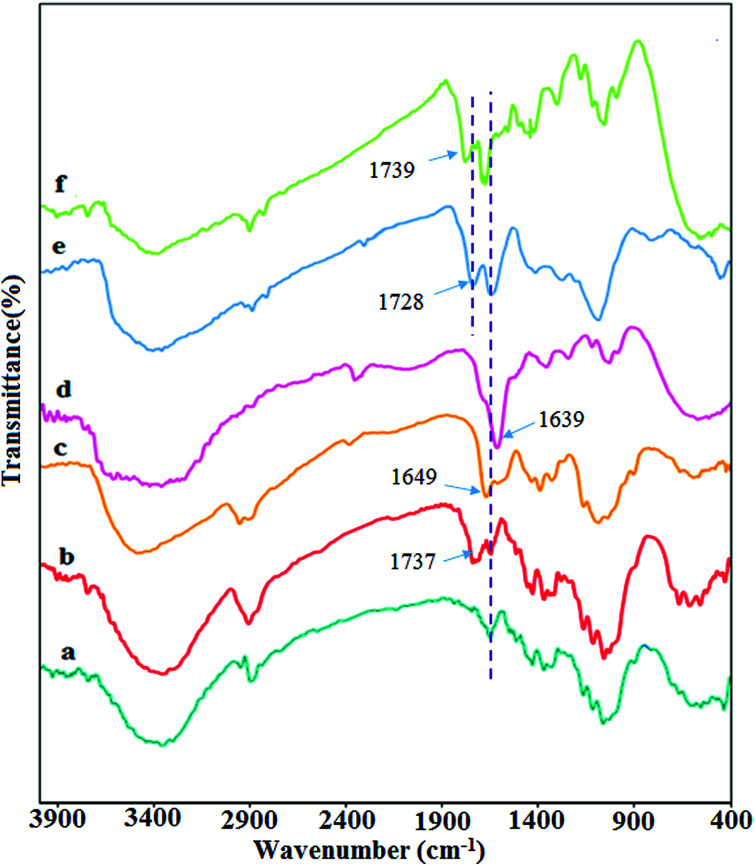
FT-IR spectra of (a) CNW, (b) DCNW, (c) chitosan NPs, (d) chitosan NPs@ DCNW, (e) GO, and (f) hydrogel.

For characterizing all of the synthesized products Fourier transform infrared (FTIR) spectroscopy was performed. Fig. 1 shows the FT-IR spectrum of all products. The FT-IR spectrum of CNW (Fig. 1a) presents the main characteristic peaks at 3400, 2900 cm^−1^ and 1000–1300 cm^−1^, which are assigned to the OH, C–H and C–O stretching vibrations, respectively. In the FT-IR spectrum of DCNW (Fig. 1b), a new absorption peak appeared at 1737 cm^−1^, which is attributed to the aldehyde groups and confirms the successful synthesis of the dialdehyde cellulose nanowhiskers. The IR spectrum of chitosan (Fig. 1c) shows the main absorption bands at 1649 cm^−1^, 1592–1613 cm^−1^, 3350–3550 cm^−1^, and 1038–1160 cm^−1^, which are assigned to the C=O stretching, NH angular deformation, OH hydroxyl group, and C–O–C in a glycoside linkage, respectively. Significant changes are observed in FT-IR spectrum of chitosan NPs@DCNW upon reaction chitosan with the dialdehyde cellulose nanowhiskers. The appearance of new peaks at 1639 cm^−1^ in the FT-IR spectrum of chitosan NPs@DCNW confirm the successful binding of chitosan to the cellulose nanowhiskers through the formation of imine bonds (Fig. 1d). The FT-IR spectrum of GO (Fig. 1e) shows stretching vibrations at 3357 cm^−1^ (assigned to O–H stretching vibrations), 1728 cm^−1^ (assigned to C=O stretching vibrations), 1627 cm^−1^ (assigned to C=C stretching vibrations) and 1028 cm^−1^ (assigned to C–O–C stretching vibrations). The IR spectrum of the hydrogel (Fig. 1f) indicates that this hydrogel is combined with the chitosan NPs@DCNW and GO. Furthermore, the shift in the peak of graphene and chitosan NPs@DCNW and the results obtained from the SEM image (Fig. 6) confirm the successful connection of graphene with chitosan NPs@DCNW within the hydrogel.

**Fig. 2:**
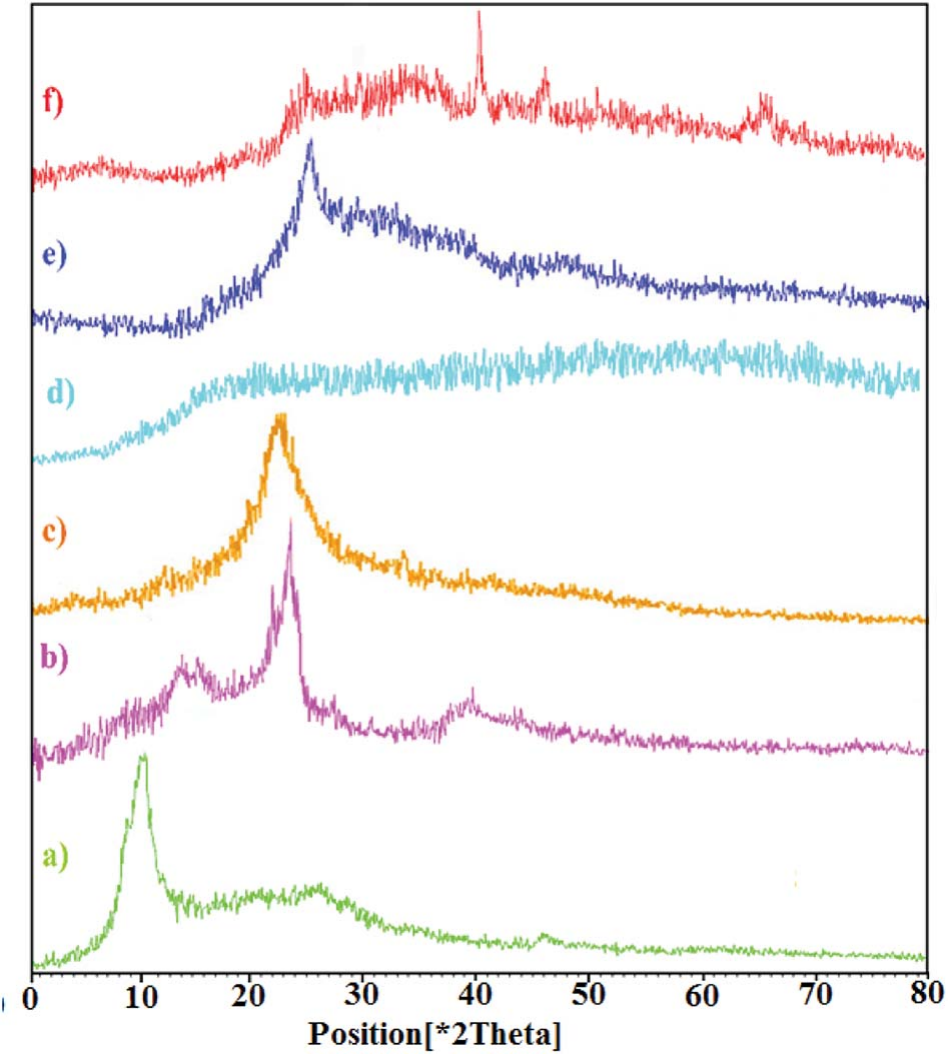
XRD patterns of (a) GO, (b) CNW, (c) chitosan, (d) chitosan NPs, (e) hydrogel, and (f) hydrogel@Pd.

The crystal structures of graphite and graphene oxide, CNW, chitosan, chitosan NPs, hydrogel, and hydrogel@Pd samples were analyzed *via* X-ray diffraction (XRD), as shown in Fig. 2. Fig. 2a shows that GO exhibits a diffraction peak at 2*θ* = 11.71° (with a *d*-spacing of 0.78 nm), corresponding to the (001) plane, which indicates that GO is fully oxidized. As shown in Fig. 2b, CNW exhibits characteristic peaks at 2*θ* values of 15.7° (101), 17.8° (101), 24.2° (002) and 38.6° (040). The high crystallinity of CNW is attributed to its intermolecular hydrogen bonds and hydrolysis of the amorphous region.^33^ In the diffractograms of the chitosan NPs (Fig. 2d), there are no peaks comparable to that of chitosan (Fig. 2c) and the broad band observed for the chitosan NPs is due to their increased amorphous nature.^34^ As shown in Fig. 2e, the XRD pattern of the nanocomposite hydrogel is different; the sharp diffraction peak of the hydrogel at 2*θ* = 11.71° disappeared and a diffraction peak appeared at 2*θ* = 24.08°. After forming the hydrogel, the strong interactions between the polymer chains and GO led to the uniform dispersion of the GO sheets in the hydrogel. This is due to the strong interactions between the polymer chains and GO. In addition, the broad peak observed for the hydrogel at 2*θ* = 24.08° is assigned to CNW. Furthermore, the XRD pattern of the hydrogel@Pd (Fig. 2f) shows four peaks at around 2*θ* = 24. 08°, 40°, 46.74°, and 67.93°, where the three peaks at 2*θ* = 40°, 46.74°, and 67.93° correspond to the (111), (200) and (220) crystal planes of Pd nanoparticles, respectively. All the peaks correspond well with those for the palladium NPs and confirm the formation of Pd(0) particles in the hydrogel. As mentioned above, the peak observed at 2*θ* = 24.08° is assigned to the presence of CNW in the hydrogel.

**Fig. 3:**
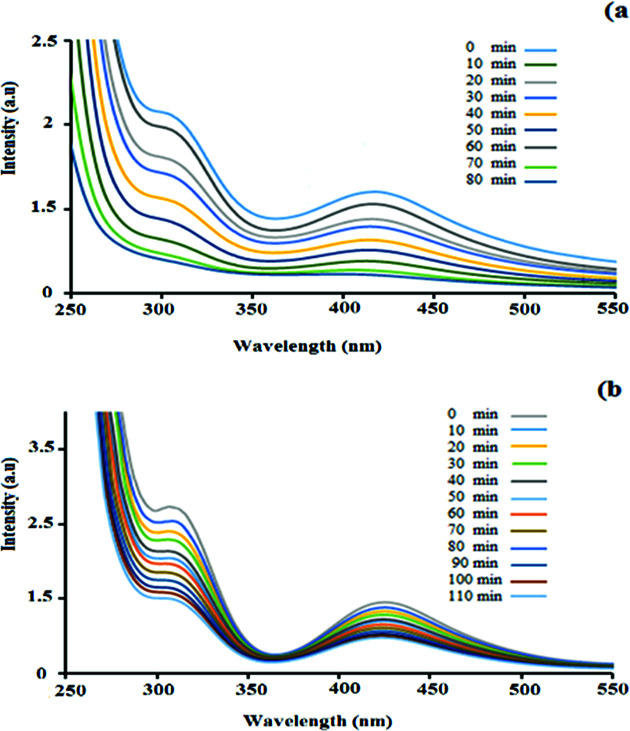
UV-vis spectra of a constant amount of the hydrogel with (a) 0.5 g PdCl_2_ in 50 mL H_2_O and (b) 0.7 g PdCl_2_ in 70 mL H_2_O. Each spectrum was collected 10 min after stirring the solution.

UV-Vis spectroscopy analysis was performed to investigate the adsorption of palladium by the hydrogel. Fig. 3 shows the UV-Vis absorbance spectra of the PdCl_2_ aqueous solution in the presence of the nanocomposite hydrogel. The PdCl_2_ solution shows strong absorption peaks at 421 nm and 306 nm, which correspond to the ligand-to-metal charge–transfer transition between the Pd^2+^ and Cl^−^ ions.^35,36^ It can be seen that with an increase in the hydrogel exposure time, the intensity of the absorption band at 421 nm and 306 nm decreased. During 80 min of stirring for PdCl_2_ (0.5 g in 50 mL H_2_O), the intensity of the absorption peak decreased and finally it disappeared (Fig. 3a). This experiment was repeated for higher concentrations (0.7 g in 70 mL H_2_O), but the amount of adsorbed Pd did not increase with the increase in concentration (even with more time) (Fig. 3b). The hydrogel weight before and after the adsorption of palladium was 0.5 g and 0.94 g, respectively.

**Fig. 4:**
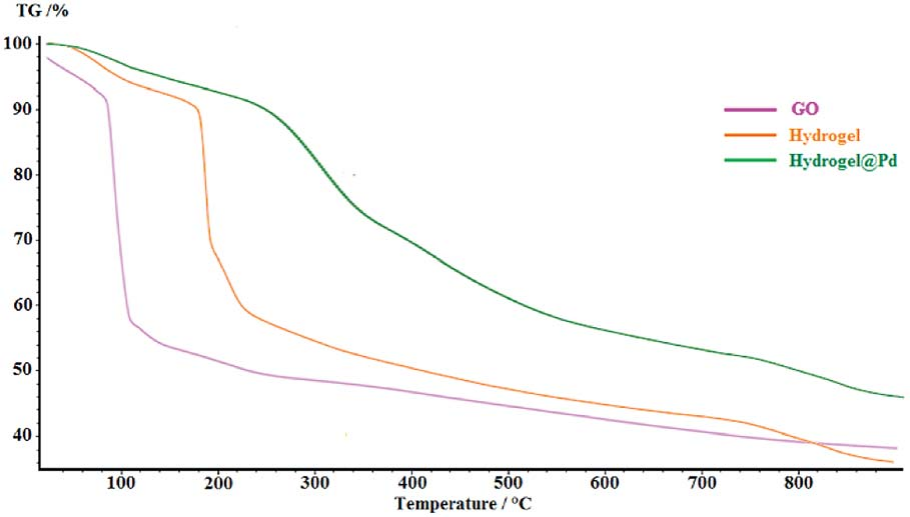
TGA thermograms of GO, hydrogel and hydrogel@ Pd.

The thermal degradation processes of GO, hydrogel and hydrogel@Pd were investigated *via* TGA, as shown in Fig. 4. The degradation amounts and the degradation temperatures (in brackets) for GO are 12 wt% (below 100 °C) and 42.3 wt% (at about 150–350 °C), which represent the removal of water and the decomposition of the oxygen-containing groups, respectively. Compared with GO, the thermal stability of the hydrogel was higher. The hydrogel exhibited three steps of mass loss with an increase in temperature. Initially, the mass loss of 7.81 wt% at 150 °C is due to the removal of H_2_O. In the second step, the severe weight loss (26.48 wt%) at about 190–210 °C can be attributed to the decomposition of the covalent and the hydrogen bonds between the compounds and decomposition of oxygen-containing functional groups on GO. Finally, the large weight loss (32.89%) at about 250–800 °C can be attributed to the decomposition of the chitosan NPs and CNW and the combustion of the carbon skeleton in GO. As can be seen in the figure, the thermal stability of the Pd nanoparticle-containing hydrogel was higher than that of the bare hydrogel since the attachment of Pd nanoparticles to the surface of the hydrogel increased its decomposition temperature. The TGA curve of hydrogel@Pd NPs exhibits higher thermal stability with a much lower mass loss up to 800 °C.

**Fig. 5:**
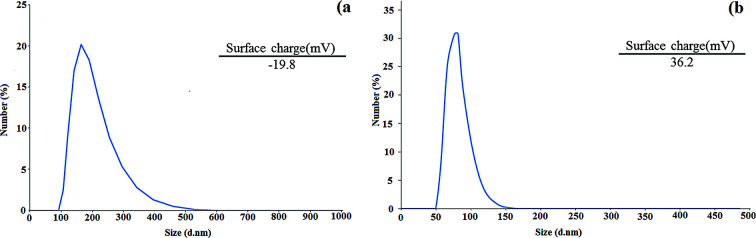
DLS diagram of CNW (a) and chitosan NPs (b).

To obtain the size distribution and particle size of the CNW and chitosan NPs, DLS measurements were performed. According to the results, the sizes of CNW and chitosan NPs are 164 and 83.8 nm, respectively (Fig. 5a and b). In addition, zeta-potential analysis was used to determine the surface charge of CNW and chitosan NPs. As shown in Fig. 5, the surface charge of CNW was −19.8 due to the presence of negatively charged hydroxyl functional groups. The zeta potential of the chitosan NPs was 36.2 due to the presence of amine groups with a positive surface charge on the nanoparticles.

**Fig. 6:**
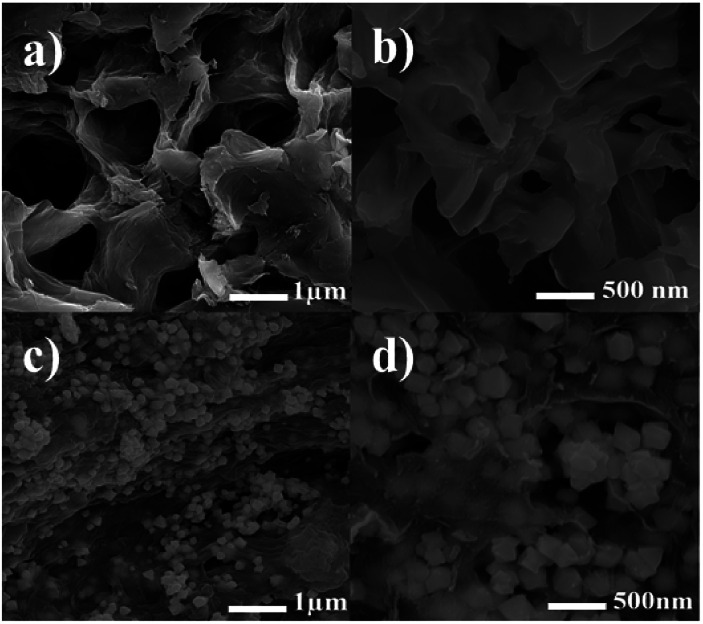
FE-SEM images of (a and b) hydrogel and (c and d) hydrogel@Pd.

The structure and morphologies of the as-synthesized hydrogel and hydrogel@Pd NPs were observed *via* FE-SEM, and the results at different magnifications are shown in Fig. 6. As can be seen clearly in Fig. 6a and b, the hydrogel consists of a macroporous and interconnected three-dimensional network.

The 3D porous structure can be very functional because it can facilitate loading and release as well as provide more void spaces. Clearly, the porous structure is ideal for transfer and mass diffusion during catalytic reactions.

Interestingly, the interconnection between pores is attributed to the network formed by the cross-linking between the chitosan NPs and dialdehyde cellulose nanowhiskers and the hydrogen bonds between the network of polymer and the GO sheets. The FE-SEM images in Fig. 6c and d show the presence of Pd nanoparticles on the surface of the hydrogel. As seen in Fig. 6d (at higher magnification), the palladium nanoparticles fill the pores and free space between the layers in the hydrogel.

**Fig. 7:**
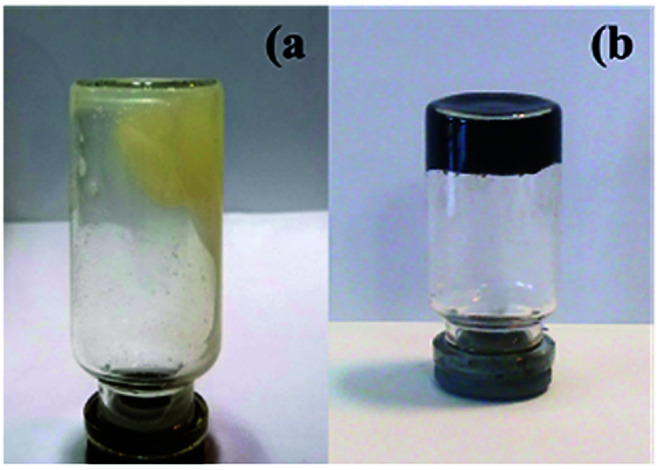
Photographs of (a) chitosan NPs@DCNW and (b) hydrogel@Pd.

As shown in the digital photos in Fig. 7a, the mixture of chitosan and DCNW polymer in water is viscous before the formation of the hydrogel. Fig. 7b shows the polymer blend containing graphene after hydrogel formation. As shown in Fig. 7, there is no hydrogel at the beginning of the experiment. However, after the addition graphene to the mixture of chitosan and DCNW polymer, due to the strong hydrogen bonding interaction formed between the polymer chains and GO, a stable hydrogel was formed from the GO nanosheets dispersed in the hydrogel. Furthermore, the hydrogel with GO exhibits a more stable network structure than the mixture without GO.

**Fig. 8:**
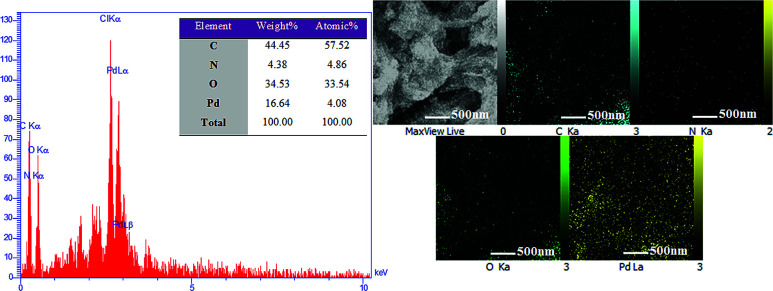
EDS spectrum of hydrogel@ Pd and elemental distribution maps in the hydrogel@ Pd region shown by EDX.

Energy dispersive X-ray spectroscopy (EDX) was used to obtain information and mass concentration of the elements available in the sample. As can be seen in Fig. 8, hydrogel@Pd contains C, N, O and Pd elements, which confirm the presence of palladium nanoparticles in the hydrogel. Furthermore, the 2D distribution maps of the Pd nanoparticles in the hydrogel matrix from EDX in Fig. 8 show that the hydrogel was homogeneously functionalized with Pd NPs.

In this study, a catalytic hydrogel containing Pd nanoparticles was designed. The catalytic properties of the hydrogel@Pd catalyst were tested *via* the Mizoroki–Heck coupling reaction. To develop a good catalytic system, the amount of catalyst, and influence of solvent and base on the Mizoroki–Heck coupling were accurately probed under reflux conditions using the reaction of iodobenzene and methyl acrylate as the model reaction (Table 1). A thorough literature survey showed no reference concerning the use of metal nanoparticles on a hydrogel support as a catalyst for C–C coupling reactions.

**Table 1:** Optimization of reaction temperature for the model coupling reaction^a^

Entry	Pd (mol%)	Solvent		Base	Time (min.)		Yield (%)^b^
1	0.20		Toluene	K_2_CO_3_		60	75
2	0.20		DMF	K_2_CO_3_		50	79
3	0.20		EtOH	K_2_CO_3_		40	80
4	0.20		H_2_O	K_2_CO_3_		60	60
5	0.20		EtOH/H_2_O	K_2_CO_3_		35	96
6	0.20		EtOH/H_2_O	NaOAc		60	77
7	0.20		EtOH/H_2_O	NEt_3_		60	65
8	0.20		EtOH/H_2_O	NaHCO_3_		65	85
9	0.20		EtOH/H_2_O	*t*-BuOK		50	79
10	0.20		EtOH/H_2_O	No base		100	Trace
11	0.15		EtOH/H_2_O	K_2_CO_3_		35	83
12	0.10		EtOH/H_2_O	K_2_CO		40	75
13	0.25		EtOH/H_2_O	K_2_CO		30	90
14	0.0		EtOH/H_2_O	K_2_CO		120	0.0

a Reaction conditions: iodobenzene, methyl acrylate, catalyst, base and solvent.

b Isolated yield.

Initially, to evaluate the effect of the catalyst, the model reaction was performed in the absence and presence of different amounts of hydrogel nanocatalyst under reflux conditions. As expected, no product was obtained in the absence of the catalyst (entry 14); therefore, the addition of a catalyst to the mixture reaction is required. The highest yield was obtained using 0.20 g of hydrogel@Pd (entry 5).

Subsequently, the reaction yield did not improve by increasing the amount of catalyst (entry 13). Furthermore, Table 1 indicates that decrease in the amount of the catalyst (entries 11 and 12) decreases the yield. Moreover, the reaction was performed using various bases in the presence of the same quantity of catalyst. As shown in Table 1, the highest yield was obtained with K_2_CO_3_ as the base in the model reaction.

In addition to the amount of catalyst and type of base, the effect of various solvents was investigated. In this regard, several solvents were selected for comparison. The results presented in Table 1 (entry 5) show that among the tested solvents, EtOH/H_2_O with a 1 : 1 ratio yielded better results than the other solvents. Aqueous ethanol was utilized as the reaction medium for economic advantage and environmental protection. Under the optimized conditions, iodobenzene, bromobenzene and chlorobenzene reacted actively with olefins. Table 2, (entries 1–12) summarizes the results obtained from this study. The structure of the compounds was compared to authentic samples and confirmed them based on their spectroscopic data.

**Table 2:** Mizoroki–Heck reaction coupling reaction of different aryl halides with olefins^a^

Entry		Olefin	X	R_1_	Time (min)	Yield (%)^b^
1	Styrene		I	H	30	98
2	Methyl acrylate		I	H	35	96
3	Methyl acrylate		I	NH_2_	40	92
4	Methyl methacrylate		I	H	35	95
5	Acrylonitrile		I	H	30	99
6	Methyl methacrylate		Br	H	55	95
7	Styrene		Br	H	40	97
8	Acrylonitrile		Br	H	45	95
9	Acrylonitrile		Br	NH_2_	65	90
10	Methyl acrylate		Br	H	60	93
11	Styrene		Cl	H	120	87
12	Methyl methacrylate		Cl	H	180	81

a Reaction conditions: iodobenzene, methyl acrylate, catalyst, base and solvent.

b Isolated yield.

After optimizing the reaction conditions, to investigate the scope and generality of this protocol, different groups of olefins and aryl halides were used in the Mizoroki–Heck reaction. As shown in Table 2, the various functional groups on aryl halides and olefins in the Mizoroki–Heck reaction can influence the rate of the reaction. Electron-rich substituents (such as NH_2_) on aryl halides cause a decrease in the reaction rate, and conversely, electron-withdrawing substituents on aryl halides increase the reaction rate (entries 3 and 9). Also, according to the data in Table 2, aryl bromides and aryl chloride produce the desired products in a longer time than aryl iodides. The increase in the length of the carbon–halogen bond in the aryl iodide results in easier metal penetration and cleavage of the bond, which is the reason for this difference. In the study of coupling reactions for catalytic systems, it was found that only iodine and bromine are active in the Mizoroki–Heck reaction with palladium, and aryl chloride is usually inactive. Fortunately, the study of hydrogel@ Pd reveals appropriate results for aryl chlorides with proper yields.

**Scheme 2:**
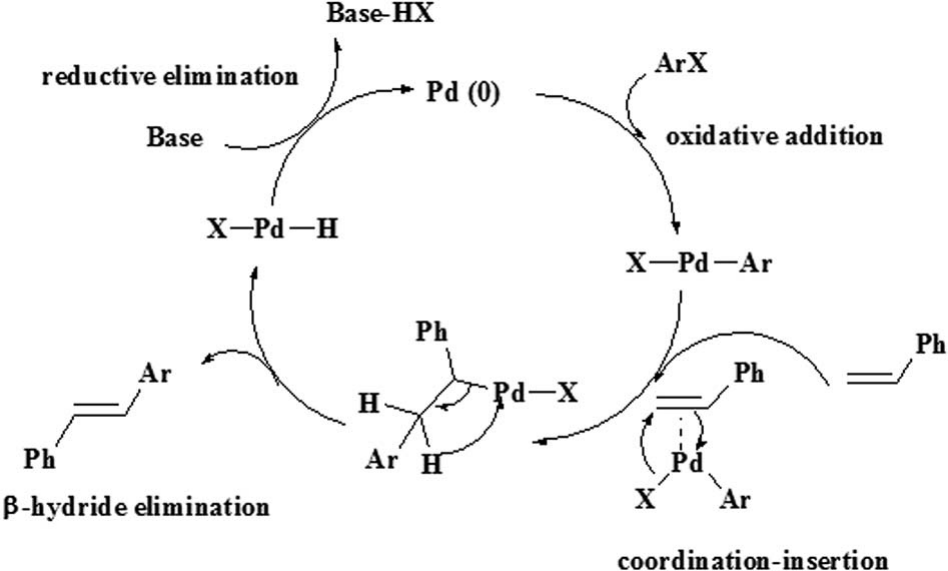
Proposed mechanism for the Heck reaction catalyzed by hydrogel@Pd NPs.

The aryl halides react with a variety of aromatic and aliphatic olefins that have electron donating and withdrawing groups. The observations in Table 2 show that olefins with electron-withdrawing groups relative to electron-donating groups have higher activity and shorter reaction times.^37^ The Heck reaction catalytic process can be explained by a plausible mechanism, as shown in Scheme 2.

The catalytic activity of hydrogel@Pd was compared to that of various Pd catalysts in the Mizoroki–Heck coupling reaction, and the results are presented in Table 3.

**Table 3:** Comparative study of the performance of various catalytic systems in the coupling reaction of chlorobenzene, bromobenzene and iodobenzene with olefins

Entry	Catalyst	Conditions	X	Time (h)	Yield (%)	Ref.
1	Pd(0) on poly(NIPAM-co-4-VP)	Bu_3_N, H_2_O, 90 °C	I	10	90	38
2	CPTH-PdNPs	NMP, Na_2_CO_3_, 80 °C	Br, I	3, 2	96, 92	39
3	Fe_3_O_4_@OA-Pd	*n*-Pr_3_N, solvent free	Cl, Br	12, 7	81, 96	40
4	Pd/WO_3_	Na_2_CO_3_, 120 °C	Br, I	5	85,96	41
5	PdLn@β-CD	K_2_CO_3_, H_2_O, reflux	Br, I	7,4	91, 94	23
6	Pd/CoBDC	Et_3_N, DMA, 90 °C	I	9	92	42
7	Seven-member analogs [(C C)PdCl_2_]	NMP, K_2_CO_3_, 130 °C	Cl, Br	24,24	71,76	43
8	Hydrogel@Pd	K_2_CO_3_, EtOH/H_2_O, 85 °C	Cl, Br, I	2, 0.66 , 0.5	87,97,98	This study

Reusability is important for heterogeneous catalysts owing to the interest in green chemistry and development of environmentally benign processes. Thus, the recycling performance of the hydrogel catalyst in the reaction of iodobenzene and methyl acrylate was also investigated. After completion of the reaction, hydrogel@Pd was separated by filtration of the reaction mixture and dialyzed with H_2_O for 3 h, and then dried under vacuum at 80 °C for 2 h. Then, it was directly used for the next reaction cycle without further purification.

**Fig. 9:**
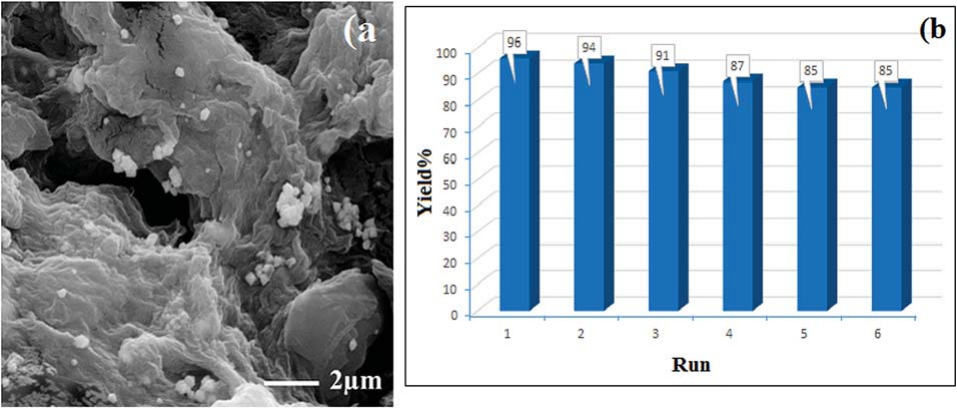
(a) FE-SEM image and (b) reusability of hydrogel@Pd for the model reaction.

After the synthesis of hydrogel@Pd, the amount of Pd NPs was determined to be 21.57 wt% by ICP-OES analysis. After six runs, the amount of Pd leached was determined to be 18.78 wt%, demonstrating the stability of the catalyst during the reaction. In addition, after a catalyst reuse period of six runs, SEM analysis of the catalyst indicated no detectable changes in the catalyst during the recovery steps (Fig. 9). Interestingly, these results demonstrate the excellent reusability of this catalyst.

## Conclusions

In the present study, a hydrogel containing Pd nanoparticles as a catalyst was successfully prepared and characterized using various techniques. The hydrogel catalyst exhibited decent reactivity and reusability in the Mizoroki–Heck reaction of aryl halides with olefins. The catalyst can be easily recovered by filtration and reused six times without significant loss in its catalytic activity and performance. This polysaccharide hydrogel is easily swollen in water solvent. It also allows the reaction to occur in greener solvents. Furthermore, the 3D structure of the hydrogel provides more space for palladium immobilization; therefore, the catalytic hydrogel results in a greater contact surface for reactions. This novel heterogeneous nanocatalyst can be widely applied to Pd-catalysed reactions.

## Conflicts of interest

There are no conflicts to declare.

## Supplementary Material

RA-008-C8RA06623J-s001
