# Supplementary material for: Preparation of a novel palladium catalytic hydrogel based on graphene oxide/chitosan NPs and cellulose nanowhiskers
Source: RSC Adv. 2018 Sep 24;8(57):32877–85. doi: 10.1039/c8ra06623j (PMC9086392; doi:10.1039/c8ra06623j)
Supplement: RA-008-C8RA06623J-s001 [file RA-008-C8RA06623J-s001.pdf]

Electronic Supplementary Material (ESI) for RSC Advances.
This journal is © The Royal Society of Chemistry 2018

Preparation a novel palladium catalytic hydrogel based on graphene oxide/ chitosan NPs and cellulose nanowhiskers biopolymers

Samira Ashiri[a], Ebrahim Mehdipour[a]*

Department of Chemistry, Faculty of Science, Lorestan University, Khoramabad, Iran.
* Corresponding Author: E. Mehdipour; E-mail: mehdipour.e@lu.ac.ir; Tel-Fax: +98 66 331206.

Since the palladium nanoparticles were synthesized in-situ in the hydrogel, there was no possibility of obtaining DLS analysis for palladium nanoparticles, and the only way to measure was SEM measurement software. One of SEM images containing the particle size, shown in Fig. S1a. The size of the nanoparticles was about 100-128 nm by SEM measurement software. Also shown in the Fig.1Sb synthesized Palladium nanoparticles have an octahedral and multifaceted structure, as a result, we can say that more surfaces are available and increased catalytic activity.

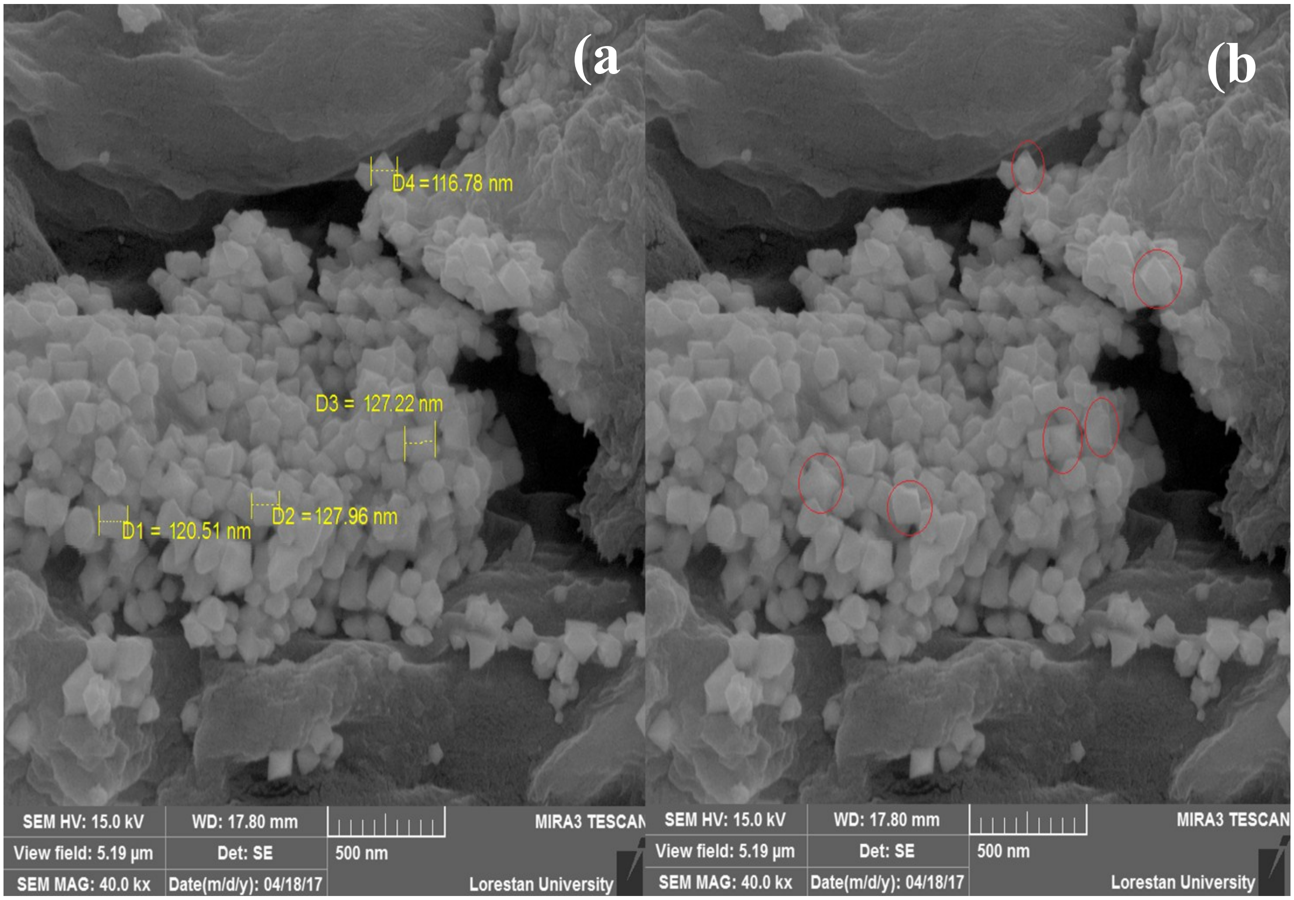

S1: FE-SEM images Palladium NPs in the hydrogel a) The size of the nanoparticles by SEM measurement software, b) Palladium nanoparticle octahedral and multifaceted structure

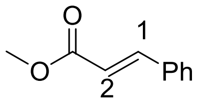
O
1
Ph
O
2

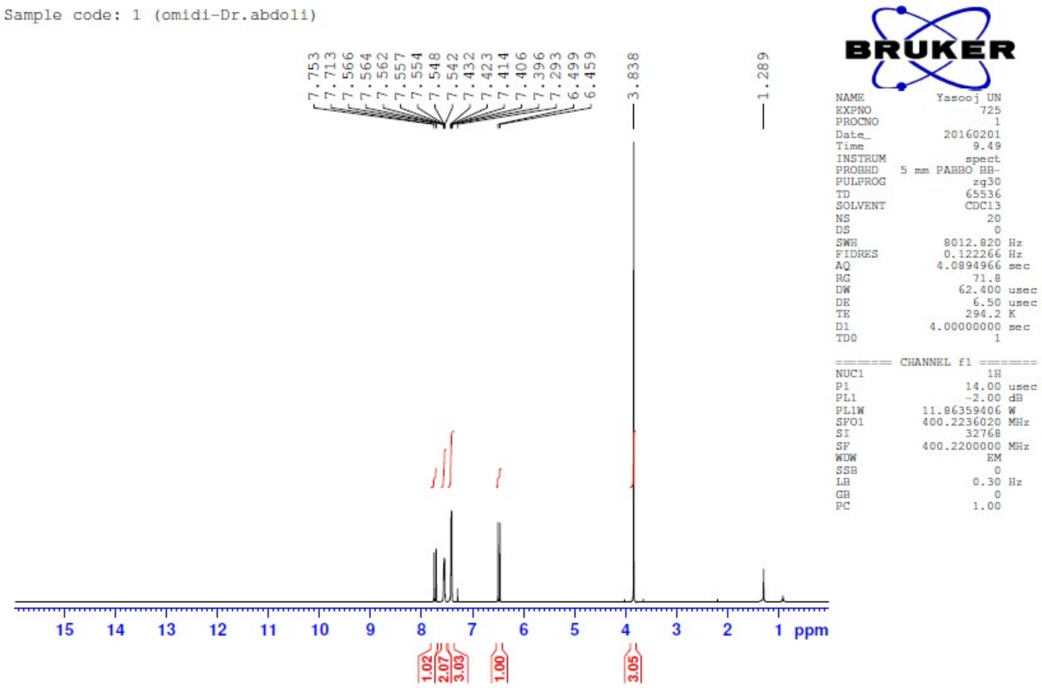
Sample code: 1 (omidi-Dr.abdoli)
7.753
7.713
7.566
7.564
7.562
7.557
7.554
7.548
7.542
7.432
7.423
7.414
7.406
7.396
7.293
6.499
6.459
3.838
1.289
BRUKER
NAME Yasooj UN
EXPNO 725
PROCNO 1
Date_ 20160201
Time 9.49
INSTRUM spect
PROBHD 5 mm PABBO BB-
PULPROG zg30
TD 65536
SOLVENT CDCl3
NS 20
DS 0
SWH 8012.820 Hz
FIDRES 0.122266 Hz
AQ 4.0894966 sec
RG 71.8
DW 62.400 usec
DE 6.50 usec
TE 294.2 K
D1 4.00000000 sec
TD0 1
====== CHANNEL f1 ======
NUC1 1H
P1 14.00 usec
PL1 -2.00 dB
PL1W 11.86359406 W
SFO1 400.2236020 MHz
SI 32768
SF 400.2200000 MHz
WDW EM
SSB 0
LB 0.30 Hz
GB 0
PC 1.00
15 14 13 12 11 10 9 8 7 6 5 4 3 2 1 ppm
1.02
2.07
3.03
1.00
3.05

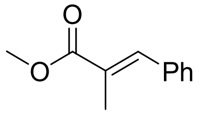
O
O
Ph

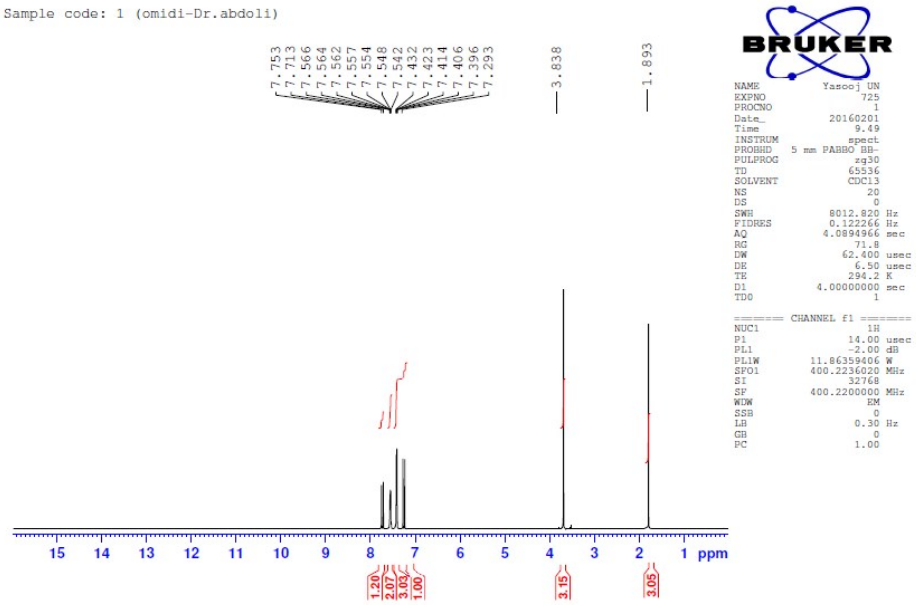
Sample code: 1 (omidi-Dr.abdoli)
7.753 7.713 7.566 7.564 7.562 7.557 7.554 7.548 7.542 7.432 7.423 7.414 7.406 7.396 7.293
3.838
1.893
BRUKER
NAME Yasooj UN
EXPNO 725
PROCNO 1
Date_ 20160201
SOLVENT CDCl3
ppm
1.20 2.07 3.03 1.00
3.15
3.05

24

25 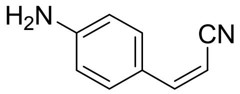
H2N
CN

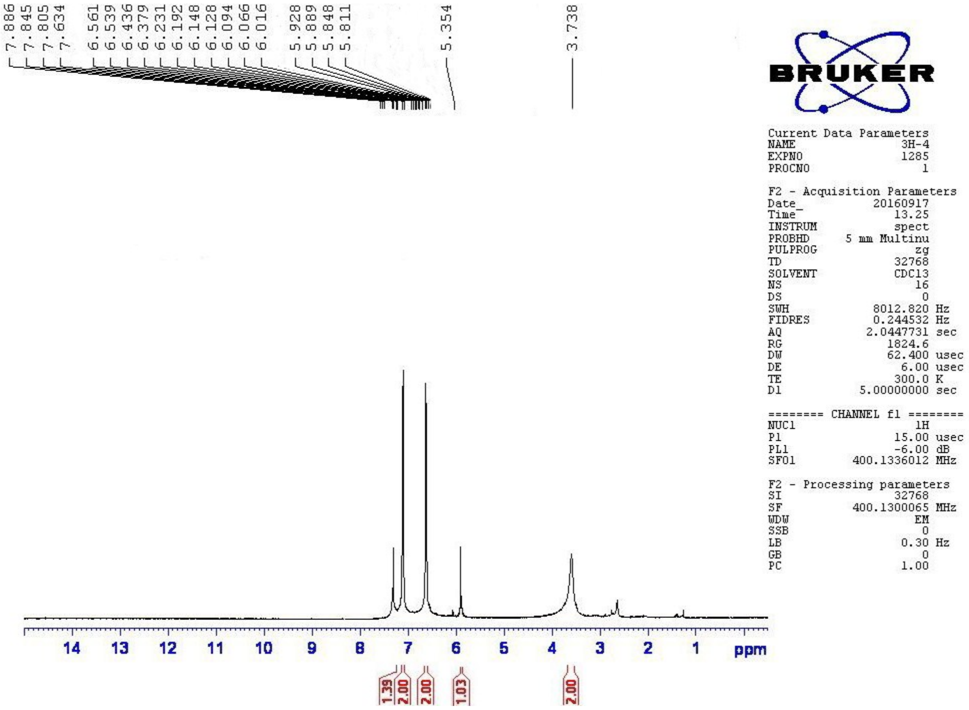
7.886 7.845 7.805 7.634 6.561 6.539 6.436 6.379 6.231 6.192 6.148 6.128 6.094 6.066 6.016 5.928 5.889 5.848 5.811
5.354
3.738
BRUKER
Current Data Parameters
NAME 3H-4
EXPNO 1285
PROCNO 1
F2 - Acquisition Parameters
Date_ 20160917
SOLVENT CDCl3
ppm
1.39 2.00 2.00 1.03
2.00

26

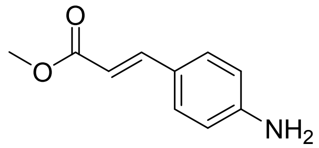
O
O
NH2

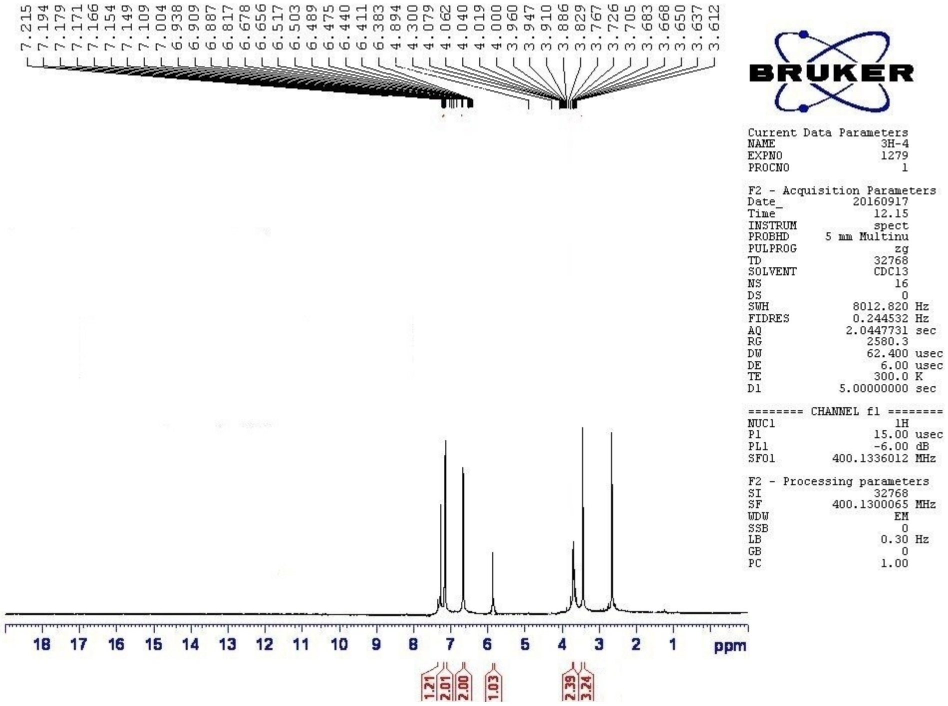
7.215
7.194
7.179
7.171
7.166
7.154
7.149
7.109
7.004
6.938
6.909
6.887
6.817
6.678
6.656
6.517
6.503
6.489
6.475
6.440
6.411
6.383
4.894
4.300
4.079
4.062
4.040
4.019
4.000
3.960
3.947
3.910
3.886
3.829
3.767
3.726
3.705
3.683
3.668
3.650
3.637
3.612
Current Data Parameters
NAME 3H-4
EXPNO 1279
PROCNO 1
F2 - Acquisition Parameters
Date_ 20160917
Time 12.15
INSTRUM spect
PROBHD 5 mm Multinu
PULPROG zg
TD 32768
SOLVENT CDCl3
NS 16
DS 0
SWH 8012.820 Hz
FIDRES 0.244532 Hz
AQ 2.0447731 sec
RG 2580.3
DW 62.400 usec
DE 6.00 usec
TE 300.0 K
D1 5.00000000 sec
======== CHANNEL f1 ========
NUC1 1H
P1 15.00 usec
PL1 -6.00 dB
SFO1 400.1336012 MHz
F2 - Processing parameters
SI 32768
SF 400.1300065 MHz
WDW EM
SSB 0
LB 0.30 Hz
GB 0
PC 1.00
18 17 16 15 14 13 12 11 10 9 8 7 6 5 4 3 2 1 ppm
1.21
2.01
2.00
1.03
2.39
3.24

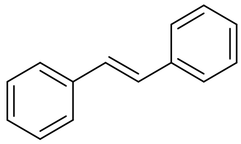

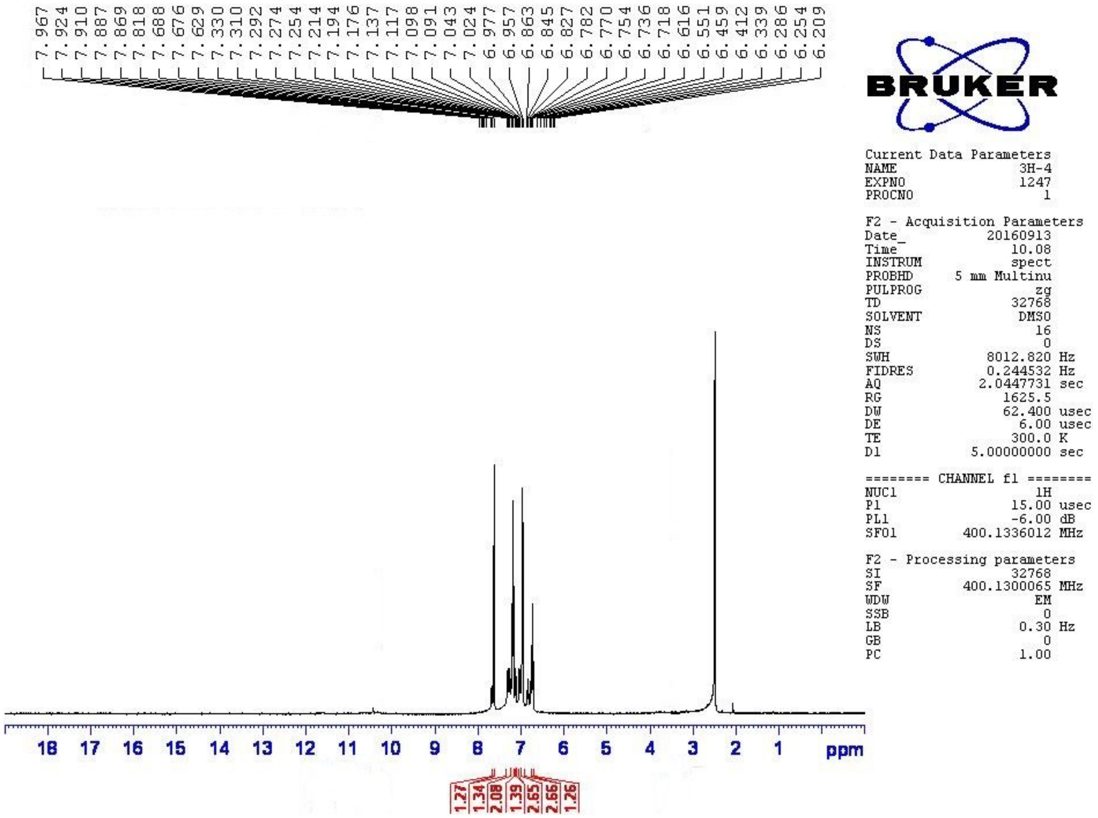

7.967 7.924 7.910 7.887 7.869 7.818 7.688 7.676 7.629 7.330 7.310 7.292 7.274 7.254 7.214 7.194 7.176 7.137 7.117 7.098 7.091 7.043 7.024 6.977 6.957 6.863 6.845 6.827 6.782 6.770 6.754 6.736 6.718 6.616 6.551 6.459 6.412 6.339 6.286 6.254 6.209
BRUKER
Current Data Parameters
NAME 3H-4
EXPNO 1247
PROCNO 1
F2 - Acquisition Parameters
Date_ 20160913
Time 10.08
INSTRUM spect
PROBHD 5 mm Multinu
PULPROG zg
TD 32768
SOLVENT DMSO
NS 16
DS 0
SWH 8012.820 Hz
FIDRES 0.244532 Hz
AQ 2.0447731 sec
RG 1625.5
DW 62.400 usec
DE 6.00 usec
TE 300.0 K
D1 5.00000000 sec
======== CHANNEL f1 ========
NUC1 1H
P1 15.00 usec
PL1 -6.00 dB
SFO1 400.1336012 MHz
F2 - Processing parameters
SI 32768
SF 400.1300065 MHz
WDW EM
SSB 0
LB 0.30 Hz
GB 0
PC 1.00
18 17 16 15 14 13 12 11 10 9 8 7 6 5 4 3 2 1 ppm
1.27 1.34 2.08 1.39 2.65 2.66 1.26

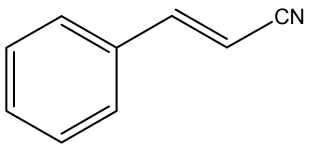
CN

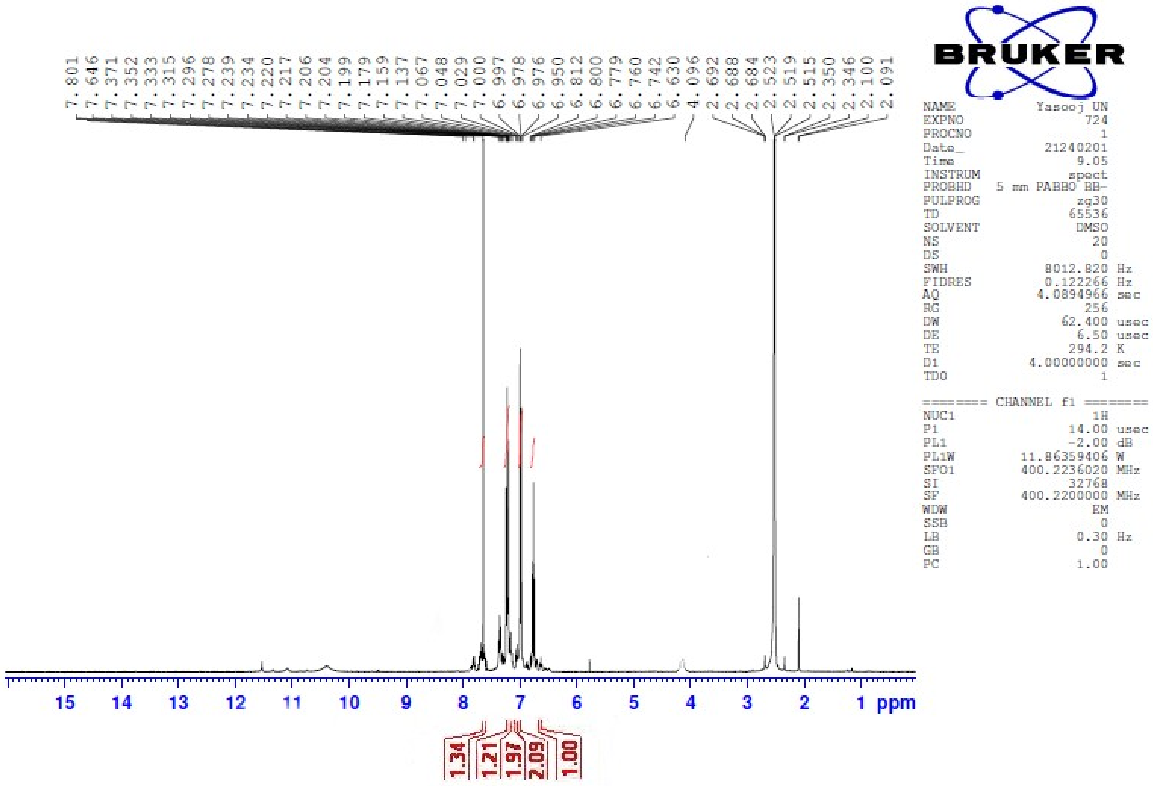
7.801 7.646 7.371 7.352 7.333 7.315 7.296 7.278 7.239 7.234 7.220 7.217 7.206 7.204 7.199 7.179 7.159 7.137 7.067 7.048 7.029 7.000 6.997 6.978 6.976 6.950 6.812 6.800 6.779 6.760 6.742 6.630 4.096 2.692 2.688 2.684 2.523 2.519 2.515 2.350 2.346 2.100 2.091
BRUKER
NAME Yasooj UN
EXPNO 724
PROCNO 1
Date_ 21240201
Time 9.05
INSTRUM spect
PROBHD 5 mm PABBO BB-
PULPROG zg30
TD 65536
SOLVENT DMSO
NS 20
DS 0
SWH 8012.820 Hz
FIDRES 0.122266 Hz
AQ 4.0894966 sec
RG 256
DW 62.400 usec
DE 6.50 usec
TE 294.2 K
D1 4.00000000 sec
TD0 1
======= CHANNEL f1 =======
NUC1 1H
P1 14.00 usec
PL1 -2.00 dB
PL1W 11.86359406 W
SFO1 400.2236020 MHz
SI 32768
SF 400.2200000 MHz
WDW EM
SSB 0
LB 0.30 Hz
GB 0
PC 1.00
15 14 13 12 11 10 9 8 7 6 5 4 3 2 1 ppm
1.34 1.21 1.97 2.09 1.00
